# Enhanced Performance of Membrane Distillation Using Surface Heating Process

**DOI:** 10.3390/membranes11110866

**Published:** 2021-11-11

**Authors:** Fei Han, Shuxun Liu, Kang Wang, Xiaoyuan Zhang

**Affiliations:** 1 School of Civil and Transportation Engineering, Hebei University of Technology, Tianjin 300401, China; a1095124986@163.com (S.L.); wangkang8012@163.com (K.W.); 2 Advanced Environmental Biotechnology Centre, Nanyang Environment & Water Research Institute, Nanyang Technological University, Singapore 639798, Singapore; Zhangxiaoyuan@tju.edu.cn

**Keywords:** membrane distillation, surface heating, hypersaline water treatment, temperature polarization, thermal efficiency, specific energy consumption

## Abstract

Membrane distillation (MD) is a thermally driven desalination process that has excellent application prospects in seawater desalination or hypersaline wastewater treatment, while severe temperature polarization (TP) and the resulting relatively high energy consumption have become principal challenges limiting the commercial application of MD. Therefore, the design of novel systems to overcome the shortage of conventional MD requires urgent attention. Here, we developed three surface heating vacuum membrane distillation systems, namely, SHVMD-1, SHVMD-2, and SHVMD-3, according to the different positions of the thermal conducting layer in the cell. The distillate flux, TP, and energy performance of these systems under different operating conditions were investigated. All three systems showed stable performance, with a salt rejection >99.98% for 35 g/L NaCl, and the highest flux was close to 9 L/m^2^·h. The temperature polarization coefficients were higher than unity in SHVMD-2 and SHVMD-3 systems, and the SHVMD-2 system produced the lowest specific energy consumption and the highest thermal efficiency. In addition, we tested the intermittent surface heating process, which can further improve energy performance through reducing specific electrical energy consumption in vacuum membrane distillation. This paper provides a simple and efficient membrane system for the desalination of brines.

## 1. Introduction

The shortage of water resources is gradually increasing worldwide [1,2,3]. The safe and efficient utilization of saline or contaminated water is of great significance for expanding water sources [4,5,6]. Generally, desalination technology can be divided into two categories according to the separation method, that is, pressure-driven membrane-based methods, represented by reverse osmosis (RO), and thermal desalination methods, represented by multi-effect distillation (MED) and multi-stage flash (MSF) [7,8,9]. Membrane distillation (MD) is a thermally driven liquid separation process. Water evaporates on the feed side and is driven by the vapor pressure and temperature difference between the two sides of the porous hydrophobic microfiltration membrane. After passing through the membrane pores, the vapor molecules are condensed and enriched at the distillate side to achieve the efficient separation of water and pollutants. MD has the advantages of simple heat transfer equipment, small footprint of equipment, strong modularity and scalability [10,11,12,13,14].

Compared with RO, MD can handle hypersaline brine as high as 180 g/L or more at atmospheric pressure and is more resistant to fouling due to its large membrane pore size [15,16,17,18].

Conventional MD requires preheating the feed liquid to generate a vapor pressure difference, but the reliance on the hot feed leads to the temperature of the membrane surface being lower than that of the feed, thus resulting in temperature polarization (TP) [19,20,21]. TP can reduce the mass transfer driving force by 40–65%, thus reducing the distillate flux and increasing the energy consumption [22,23]. Therefore, mitigating the TP is of great significance for improving MD performance in terms of distillate flux and energy consumption. Recently, a self-heated vacuum membrane distillation (VMD) system was developed, which differs fundamentally from a conventional VMD in that heat was directly supplied to the membrane surface. Directly providing heat to the membrane/water interface can mitigate TP and improve the energy efficiency of the system, for example, applying radio frequency induction to the VMD system and using induction heating to directly heat the brine at the membrane/water interface, thus providing an effective driving force for the MD process while reducing the effects of TP [24]. Coating carbon nanotubes on the polytetrafluoroethylene (PTFE) membrane to form a conductive composite layer, and applying a high-frequency alternating current to generate Joule heating, can make the temperature of the membrane surface higher than the temperature of the bulk feed, which could mitigate TP [25]. In addition, since MD can operate at low temperatures, solar heating is considered an efficient and environmentally friendly method [26,27,28]. The application of new photothermal and electrothermal materials also makes it possible to directly heat the membrane surface [29]. A double-layer hydrophilic/hydrophobic membrane composed of carbon black-loaded polyvinyl alcohol nanofibers and a polyvinylidene fluoride (PVDF) hydrophobic membrane can absorb solar radiation to heat the cold feed [30]. These methods are effective in mitigating TP, but are still limited by the velocity of and temperature decrease in the feed [28,29,30,31,32,33]. In addition, the use of novel MD process designs, such as an O-Ring VMD, could also improve the system flux [34]. Previous studies have demonstrated the feasibility and effectiveness of conducting thermal energy to the membrane/water interface [35,36,37]. However, further research such as increasing the thermal efficiency of the system and reducing energy consumption is still needed to improve system performance. Therefore, in this study, we optimized the configuration of thermal conducting layers and designed three surface heating VMD systems (namely, SHVMD-1, SHVMD-2, and SHVMD-3). We chose VMD as the basic process because there is negative pressure on the permeate side of VMD, while TP only occurs on the feed side, which can minimize heat loss for a stable flux [38]. The influence of the configuration and operating conditions of these systems on flux are discussed. In order to further reduce the energy consumption of the system, we conducted intermittent heating experiments by changing the heating conditions of the external heat source [39,40]. The systems used in this paper can reverse the adverse effects of TP, reduce energy consumption, and provide a solution for the treatment of brines by MD.

## 2. Materials and Methods

### 2.1. Materials

A hydrophobic PTFE membrane (Shengju Tech Co, Tianjin, China) with a thickness of 50 μm, pore diameter of 0.2 μm and porosity of 81% was used in this study. In addition, the size of the aluminum shim used as the thermal conducting layer of the system was 11 cm × 25 cm × 0.08 mm. We used 35 g/L NaCl solution as the feed to test the desalination performance of these three VMD systems; sodium chloride (AR grade) was purchased from Damao Reagent Co., Ltd., Tianjin, China.

### 2.2. Surface Heating System Design

**Figure 1:**
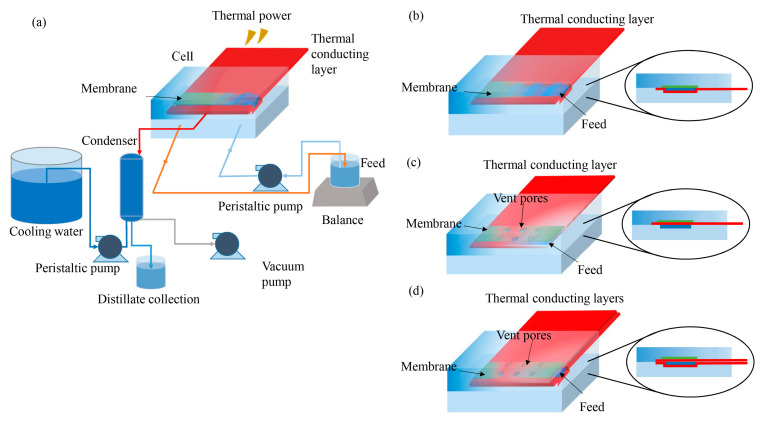
Schematic diagram of the experimental setup for (**a**) schematic of a laboratory-scale surface heating VMD system; (**b**) SHVMD-1 system: the aluminum shim was embedded into the feeding channel; (**c**) SHVMD-2 system: the aluminum shim was placed close to the membrane surface of the feed side; (**d**) SHVMD-3 system: double-layer shims, one of which was embedded into the feeding channel, and the other was placed close to the membrane surface.

The surface heating systems were designed as shown in Figure 1a. Heat was delivered to the feed flow channel or the hydrophobic membrane surface by heat conduction and convection through a metallic thermal conducting layer. An aluminum shim was chosen as the thermal conducting layer due to its good thermal conductivity, excellent mechanical strength, and low cost.

Three configurations of thermal conducting layers were studied in this paper. Connect one end of the aluminum shim to an external heat source and embed the other end of the shim into the feeding channel of the cell to compose the SHVMD-1 system (Figure 1b). Connect one end of the aluminum shim with vent pores to an external heat source and place the other end of the shim close to the membrane surface of the feed side in the cell to constitute the SHVMD-2 system (Figure 1c). Simultaneously place the aluminum shims in the SHVMD-1 and SHVMD-2 into the cell to assemble the SHVMD-3 system (Figure 1d).

### 2.3. Surface-Heating VMD Experiments

The experiments were performed using laboratory-scale VMD systems (Figure 1a), which were composed of a heat source, feeding pump, cell, vacuum, and condenser. The thermal power provides energy, and the thermal conducting layer plays the role of transferring heat into the cell. A flat sheet PTFE membrane with an effective area of 40 cm^2^ (4 cm × 10 cm) was installed in the cell. The height of the feed and distillate flow channels was 4 mm. Pieces of aluminum (Al) shim or Al shim with vent pores were used as the thermal conducting layer. A peristaltic pump with temperature-resistant tubing circulated the feed solution, and the flow velocity was controlled by the pump controller. A vacuum pump generated a vacuum in the range of 0~100 kPa on the distillate side of the membrane.

As shown in Figure 1a, feed solution was circulated in the feed side by the peristaltic pump (Lead Fluid, BT101T, Baoding, China). The external heat source (Chang Run, XMT615, Shenzhen, China) transferred heat to the cell through the thermal conducting layer. The heat was delivered to the membrane/water interface for evaporation. Water vapor diffused through the membrane pores and entered the permeate side via a vacuum pump (Yong Hao, 2XZ-4, Linhai, China); it was then condensed by a condenser, which was driven by another peristaltic pump (Lead Fluid, BT600-2J, Baoding, China). The temperature inside the cell and flow channel was measured using thermocouples (Shenhua, Type K, Shenzhen, China). Additionally, the conductivity of feed and produced water was measured using a thunder magnetic conductivity meter (DDS-11A, Ray Magnetic, Shanghai, China). The experiment was repeated three times, and the results are reported as averages.

### 2.4. System Performance

#### 2.4.1. Distillate Flux

The distillate flux was calculated using Equation (1) [20]:

(1) $$J=\frac{\Delta m}{\rho At}$$

where *J* (L/m^2^·h) is the distillate flux; ∆*m* (kg) and *ρ* (kg/L) are the quality reduction and density of the feed solution, respectively; *A* (m^2^) is the effective membrane area; and *t* (h) is the operation time.

#### 2.4.2. Rejection

The rejection rate can be expressed in terms of the conductivity of the feed. In this experiment, a thunder magnetic conductivity meter was used to measure the conductivity as follows:

(2) $$R=\frac{\left(k_{f}-k_{p}\right)}{k_{f}}\times 100\%$$

where *R* is the rejection rate (%); *k_f_* (mS/cm) and *k_p_* (mS/cm) are the conductivities of the feed and permeate, respectively.

#### 2.4.3. Temperature Polarization Coefficient

The temperature polarization coefficient (TPC) was used to quantify the severity of TP, which can be defined by the ratio of the membrane surface temperature on the feed side to the temperature of the bulk feed in the VMD system, and can be calculated as follows:

(3) $$\mathrm{TPC}=\frac{T_{mf}}{T_{bf}}$$

where *T_mf_* (°C) is the membrane surface temperature on the feed side; *T_bf_* (°C) is the bulk temperature of the feed.

#### 2.4.4. Heat Transfer Performance

The surface heating VMD does not need to preheat the feed solution but requires transfer heat to the membrane/water interfaces through the thermal conducting layer. In the heat transfer process, the external heat source conducts heat to the heat conducting layer, then convective heat exchange occurs between the heat conducting layer and the feed. The convective heat exchange that occurs in the vacuum can usually be ignored [40,41,42,43,44].

*Q_in_* (W) is the transfer of heat from the heat source to the thermal conducting layer, which can be calculated using the following Equation:

(4) $$Q_{in}=A_{m}K_{m}\frac{\Delta T}{d}$$

where *A_m_* is the cross-section area of the shim (m^2^); *K_m_* is the heat transfer coefficient of the aluminum shim, 237 W/m·K; *d* (m) and Δ*T* (K) are the heat conduction distance on the shim and the temperature difference of the distance, respectively.

*Q*_v_ is the heat absorbed as latent heat when water evaporates, which can be calculated as follows:

(5) Qv=JAΔHv

where ∆*H_v_* is the latent heat of evaporation of water, 2357.6 kJ/kg.

The system thermal efficiency (TE) refers to the proportion of the total heat required for evaporation, which is calculated as follows:

(6) $$\mathrm{TE}(\%)=\frac{Q_{v}}{Q_{in}}$$

SEC (kWh/L) is defined as the amount of total energy supplied to produce a unit mass of pure water, which is calculated as in Equation (9) [37,45]:

(7) SEC=STEC+SEEC

where STEC (kWh/L) and SEEC (kWh/L) are the specific thermal energy consumption and the specific electrical energy consumption, respectively, which can be calculated as in Equations (8) and (9) [46]:

(8) $$\mathrm{STEC}=\frac{Q_{in}\rho }{JA}$$

(9) $$\mathrm{SEEC}=\frac{E\rho }{JA}h$$

where *E* (kJ/s) presents the rate of electrical energy input, including the vacuum pump, peristaltic pump, and condenser.

## 3. Results and Discussion

### 3.1. Effect of Operating Conditions on Distillate Flux

Here, we discuss the effects of the operating conditions, including the heat source temperature, crossflow velocity, vacuum, and feed salinity, on the distillate flux and the corresponding heat input of the three systems.

**Figure 2:**
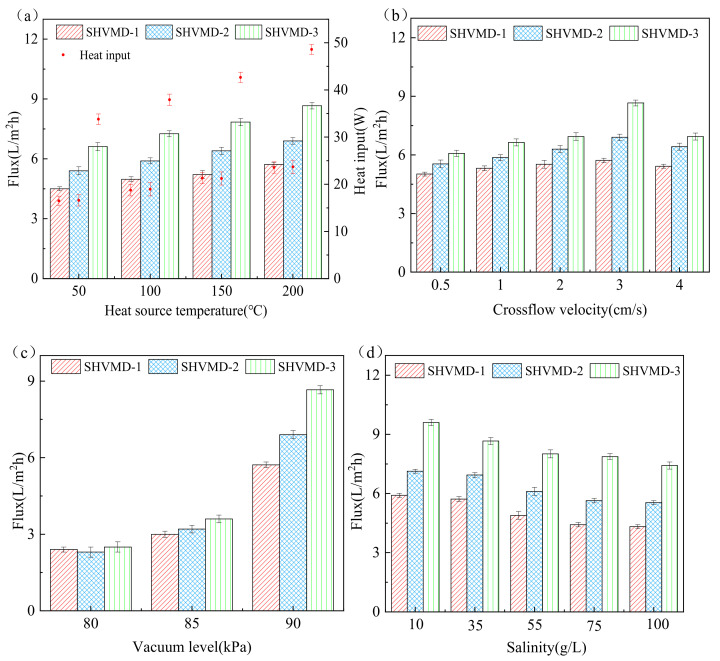
Heat input and distillate flux in 2 h tests with different system configurations. The flux and heat input of SHVMD-1, SHVMD-2, and SHVMD-3 were measured as a function of (**a**) heat source temperature, (**b**) crossflow velocity, (**c**) vacuum level, and (**d**) salinity. Regarding the operating conditions, unless specified as the variable, the heater temperature was 200 °C, crossflow velocity was fixed at 3 cm/s, vacuum level was kept at 90 kPa, and salinity was 35 g/L.

Figure 2a shows that with the increase in heat source temperature from 50 °C ± 1 °C to 200 °C ± 1 °C, the flux of the three systems increased gradually. The reason for this is that the driving force of VMD mass transfer is mainly derived from the transmembrane vapor pressure difference, which is composed of the saturated vapor pressure difference and vacuum level [21]. Under constant vacuum conditions, the transmembrane pressure difference is determined by the saturated vapor pressure difference, while the saturated vapor pressure and the surface temperature of the membrane are positively correlated according to the Antoine Equation [43]. Therefore, as the transmembrane pressure difference increases with the heat source temperature, the flux eventually increases.

The flux in the SHVMD-2 system was increased by 20% ± 1.8%, 19% ± 1.9%, 20% ± 2.3%, and 21% ± 2.1% at four different heat source temperatures, respectively, compared to that in the SHVMD-1 system. This can be attributed to the direct contact between the thermal conducting layer and the hydrophobic membrane in the SHVMD-2 system, making the membrane surface temperature higher than the bulk feed temperature, which results in the elimination of TP, the reduction in heat and mass transfer resistance, and the increase in distillate flux [31].

The input heat in all systems increased with the heat source temperature (Figure 2a). Under the same operating conditions, the flux of the SHVMD-2 system was significantly higher than that of the SHVMD-1 system, while the heat input was almost equal in the two systems, indicating that system configuration—in addition to heat input—plays an essential role. In the SHVMD-1 system, the thermal conducting layer heated the feed from the bottom of the feed channel, causing unnecessary heat loss because part of the energy was used in the non-evaporation process. However, in the SHVMD-2 system, the heat was directly transferred to the membrane/water interface, which can minimize the heat loss of the non-evaporating process. This can be attributed to the fact that evaporation is a surface process, in which water molecules at the very thin air/water interface in the feed side, driven by their high energy state, are transported into the vapor phase [42].

Although doubled heat was input in the SHVMD-3 system due to the use of a dual thermal conducting layer, the increase in flux was not considerable compared to the others, which may be related to system thermal efficiency and be discussed in the following section.

The distillate flux of the three systems increased first and then decreased with crossflow velocity (Figure 2b). The increase in flux can be interpreted as the increase in fluid velocity with the increase in volumetric flow rates; thus, the convective heat transfer coefficient developed, and the thermal boundary layer thickness decreased. The subsequent decrease in flux can be explained as the residence time of the feed solution becoming shorter, and the high crossflow velocity led to a decrease in heat absorbed by the feed in the cell, resulting in a decrease in distillate flux. In addition, high crossflow velocity may lead to an increase in pressure on the membrane surface, increasing the risk of membrane wetting [47]. Therefore, the velocity used in this study was 3 cm/s.

**Figure 3:**
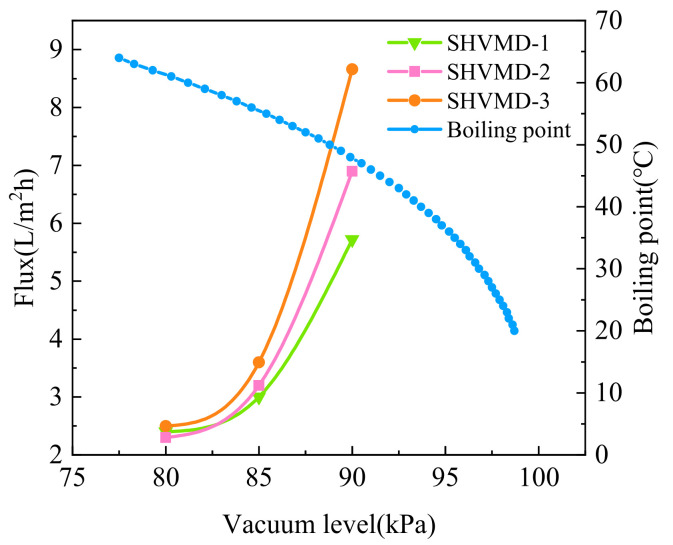
Changes in flux and boiling point of solution with the vacuum level.

The distillate flux increased with the vacuum level, indicating that vacuum level had a great influence on the transmembrane pressure difference in this study (Figure 2c). An ideal flux performance was obtained by combining the suitable temperature and vacuum level. The flux was only slightly changed with a vacuum level of less than 85 kPa; then, the flux increased sharply. This is because the driving force of mass transfer of VMD is mainly derived from the transmembrane pressure difference, which is composed of vacuum level and saturated vapor pressure differences [41]. Thus, a greater mass transfer driving force was obtained via an increase in the vacuum level at a certain heat source temperature. In addition, according to the Clausius–Clapeyron equation, the boiling point changes non-linearly with the vacuum level [48]. The increase in the vacuum level can greatly reduce the boiling point, thereby increasing the distillate flux (Figure 3).

When the salinity increased from 10 g/L to 100 g/L, the distillate flux decreased because the partial pressure of water vapor on the membrane surface decreases with the salinity, thus reducing the transmembrane pressure difference. However, the flux still reached 7 L/m^2^·h at 100 g/L salinity (Figure 2d), indicating that the systems were not sensitive to salinity. As the salinity of the solution in the thermal boundary layer on the surface of the hydrophobic membrane gradually increased with the evaporation process of the feed solution, concentration polarization occurred. When the concentration of feed near the thermal boundary layer reaches saturation, salt crystals will be produced, thus resulting in membrane scaling and a decrease in the distillate flux.

All of the above operating conditions had little effect on the rejection rate, which remained above 99.98%, showing the inherent advantages of MD in the treatment of brine.

### 3.2. TPC

**Figure 4:**
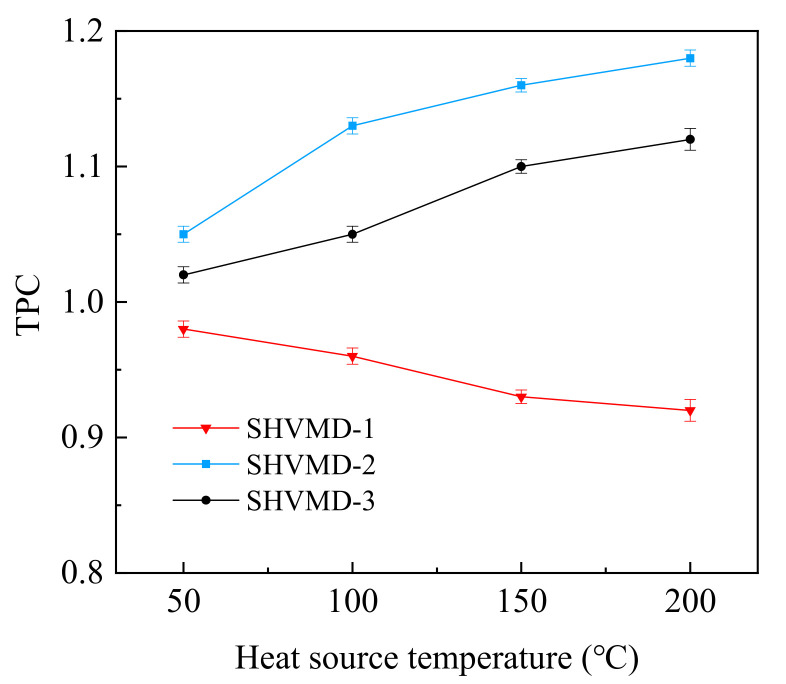
TPC changes with heat source temperature in different systems.

The TPC decreased with heat source temperature in the SHVMD-1 system because the thermal conducting layer conducted the heat to the feed, then transferred it to the membrane surface, resulting in the temperature in the feed being higher than that in the membrane surface (Figure 4). In addition, the convective heat transfer rate and flux increased with the increase in temperature [49]. Therefore, the evaporation took away more heat and reduced the temperature of the membrane surface, leading to a decrease in TPC.

However, in the SHVMD-2 and SHVMD-3 systems, the thermal conducting layer was directly in contact with the membrane surface. The heat taken away by the evaporation was supplemented to the thermal boundary layer by the thermal conducting layer, which overcame the negative impact of the reduction in the driving force at the surface of the membrane due to evaporation and heat absorption of the feed. In addition, the thermal conducting layer also played a role in heating the hydrophobic membrane. Although both the membrane surface and the feed were heated up, the circulation of the feed made it difficult to accumulate the heat transferred from the thermally conductive layer to the feed. Moreover, the hydrophobic membrane was a poor conductor of heat, which caused the membrane surface temperature to be higher than the feed temperature. Therefore, TPC gradually increased with the heat source temperature.

In contrast to the SHVMD-1 system, the TPC values of SHVMD-2 and SHVMD-3 systems both overcame the bottleneck of unity, indicating that the direct heating of the membrane surface by the thermal conducting layer can effectively alleviate the TP. Due to the double thermal conducting layer in the SHVMD-3 system, the temperature difference between the membrane surface and the feed was less than that in the SHVMD-2 system; thus, the TPC in the SHVMD-3 system was lower than that in the SHVMD-2 system.

The above results showed that both SHVMD-2 and SHVMD-3 have excellent alleviating effects on TP, and the SHVMD-2 system performed best. In addition, the TP in the MD process was not affected by a single factor but by comprehensive manifestations under multiple conditions. Therefore, in practical applications, it is necessary to balance water production and energy consumption to obtain the appropriate operating temperature.

### 3.3. Energy Performance

Energy performance is a critical restriction for the application of MD, so it is necessary to analyze the specific energy consumption and thermal efficiency. We tested the SEC and TE of the three systems at different heat source temperatures.

**Figure 5:**
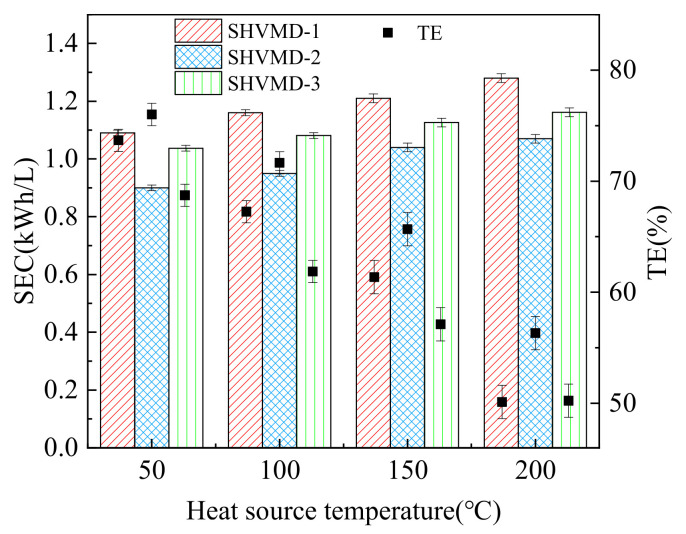
SEC and TE of the three systems under different heat source temperatures. All tests were performed with a feed salinity of 35 g/L, crossflow velocity of 3 cm/s, and vacuum level of 90 kPa.

Results showed that as the temperature of the heat source increased, the SEC in each system increased slightly, while the TE decreased significantly. The SHVMD-3 system had a higher SEC and the lowest TE (Figure 5). The reason for this is that the heat transferred into the system can be divided into three parts: one is used for evaporation, another is used to raise the temperature of the feed, and the other is lost to the environment. The temperature of the feed rises with the heat input, leading to an increase in the three parts. However, a high proportion of heat used for evaporation is required to increase TE. The high heat input leads to more heat being taken away by the hot feed circulation and the environment. This can explain why it has the highest heat input, whereas the flux was not considerable, as discussed in Section 3.1.

Conversely, the SHVMD-2 system had the lowest SEC and the highest TE. Under the same heat source temperature, the TE of the SHVMD-2 system was higher than that of the SHVMD-1 system, while the heat input was almost the same because the SHVMD-2 system could minimize non-evaporative heat loss.

### 3.4. Intermittent Surface Heating Process

We found in our study that when the heat source was turned off for a period of time, the flux only decreased slightly, and more importantly, the energy consumption of the system was reduced. Therefore, we improved the above process and proposed a new process called intermittent surface heating. For the SHVMD-2 system, we conducted experiments at heat source temperatures of 50 °C, 100 °C, and 200 °C, respectively. The SHVMD-2 system was selected because it can minimize heat loss in the intermittent surface heating process.

**Figure 6:**
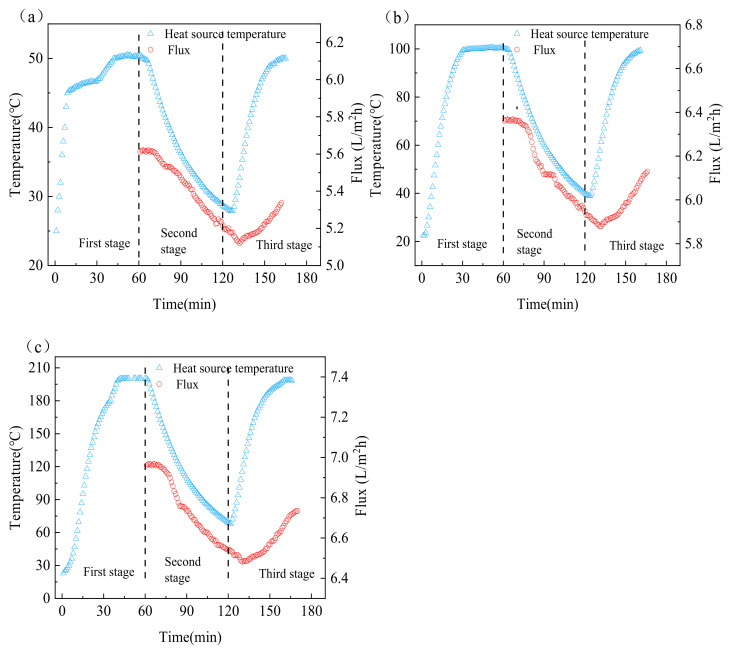
Changes in heat source temperature and flux with time in the intermittent heating process at heat source temperatures of (**a**) 50 °C, (**b**) 100 °C, and (**c**) 200 °C.

The procedure of the intermittent heating experiment can be divided into three stages. In the first stage, the system was started, and the temperature of the heat source was increased to the set temperature until stable, which required approximately 60 min. Then, heating was stopped for 60 min and, using the residual heat to maintain the operation of the system, the flux changes with time were recorded. During this time, the heat source temperature decreased by 41.3%, 60.4%, and 65.8% at the initial temperature of 50 °C, 100 °C, and 200 °C, while the flux only decreased by 8.4%, 7.4%, and 7.0%, respectively (Figure 6a–c). Although the temperature of the heat source decreased by more than a half, the flux decreased slightly, indicating that the heat transfer from the heat source to the cell can provide the driving force required for the operation of the system. This urged us to reduce energy consumption at the cost of a small amount of flux, which required verification by calculating SEC.

**Figure 7:**
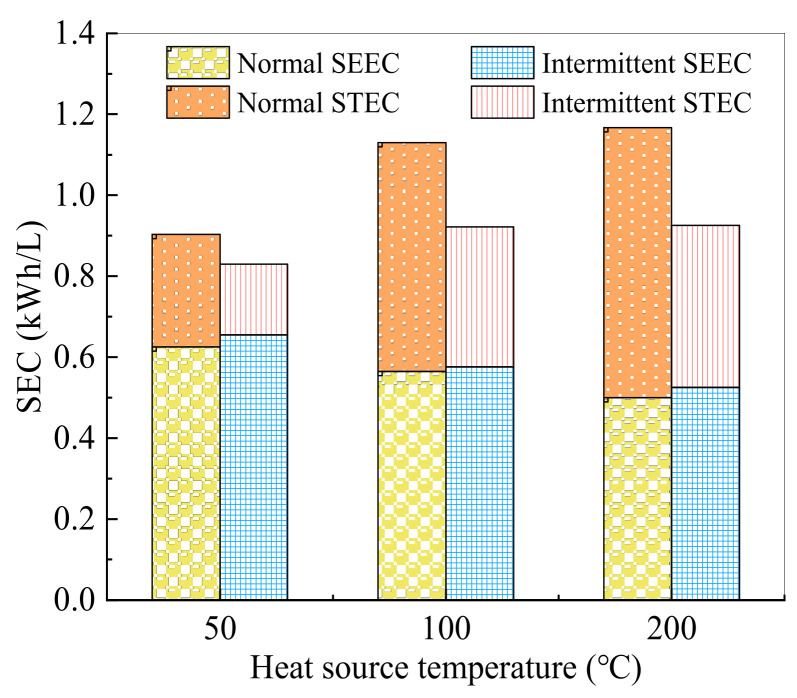
Comparison of SEC with and without intermittent heating at different heat source temperatures.

The SEC of the intermittent surface heating experiment increased with the temperature of the heat source (Figure 7), which was consistent with the results of the normal surface heating system. By subdividing SEC into SEEC and STEC, the values of SEEC decreased with the increase in the heat source temperature. This is because the high-temperature system has a high flux, and the electric energy consumed per unit of water is reduced when the electric energy consumption is basically the same. However, the STEC showed the opposite trend because the higher the heat source temperature, the more heat energy needs to be input. Although the flux increased, the required heat input increased more, which led to an increase in STEC.

For intermittent surface heating, when the heat source temperature was 50 °C, 100 °C, and 200 °C, the SEC decreased by 8.1%, 18.0%, and 20.7%, while the STEC declined by 37.1%, 38.2%, and 40%, respectively (Figure 7). Therefore, intermittent heating had a major influence on the STEC of the system, because the energy consumption was greatly reduced after turning off the heat source. In addition, the higher the heat source temperature, the better the optimization of system energy consumption by intermittent heating.

These results indicate that intermittent heating systems can reduce SEC by up to 20% at the expense of less than 10% flux. This may lead to new breakthroughs in decreasing the energy consumption of MD.

## 4. Conclusions

In this study, three surface heating vacuum membrane distillation systems were developed for the desalination of brines, and the effects of operating conditions on the system performance were also discussed. The results showed that salt rejection in all systems was above 99.98% at a feed salinity of 35 g/L, a crossflow velocity of 3 cm/s, and a vacuum level of 90 kPa. Furthermore, it was found that the surface heating could significantly reduce the TP while increasing the distillate flux. The performance of SHVMD-1 was not as high as that of SHVMD-2 and SHVMD-3 in terms of flux and energy consumption. The SHVMD-2 system was shown to be effective in eliminating the negative impact of TP on MD, and achieved the highest thermal efficiency. On the other hand, a higher flux and heat loss were observed in SHVMD-3 as compared to SHVMD-1 and SHVMD-2. In practical applications, a trade-off between flux and energy consumption should be taken into account in the selection of SHVMD-2 or SHVMD-3. Intermittent heating resulted in a flux decline of less than 10% in exchange for a reduction of up to 20% SEC, indicating the effectiveness of the intermittent heating in reducing system energy consumption. It should also be pointed out that the efficacy of intermittent surface heating in other MD processes with lower thermal efficiency still need further verification. It is expected that this study can offer useful insights into energy-efficient MD processes.

## Nomenclature

*A*	effective area of the membrane, m^2^
*A_m_*	cross-section area of the shim, m^2^
*C*	specific heat capacity, J/kg·°C
*d*	heat conduction distance on the shim, m
*E*	rate of electrical energy input, kJ/s
*∆H_v_*	latent heat of evaporation, kJ/kg
*J*	permeate flux, L/m^2^·h
*k_f_*	conductivity of the feed, mS/cm
*k_p_*	conductivity of the permeate, mS/cm
*K_m_*	coefficient of thermal conductivity, W/m·K
*∆m*	mass reduction in feed, kg
*Q_in_*	total heat transfer to the system, W
*Q_v_*	heat transfer across the VMD, W
*R*	rejection rate, %
SEC	specific energy consumption of the system, kWh/L
SEEC	specific electrical energy consumption, kWh/L
STEC	specific thermal energy consumption, kWh/L
*t*	operation time, h
*ΔT*	temperature difference of the distance, K
*T_bf_*	bulk temperature of the feed, °C
*T_mf_*	membrane surface temperature on feed side, °C
TE	thermal efficiency, %
TPC	temperature polarization coefficient
*ρ*	density of feed solution, kg/L
